# The use of an alternate side lying positioning strategy during inhalation therapy does not prolong nebulisation time in adults with Cystic Fibrosis: a randomised crossover trial

**DOI:** 10.1186/s12890-017-0568-2

**Published:** 2018-01-08

**Authors:** Ruth L. Dentice, Mark R. Elkins, Genevieve M. Dwyer, Peter T. P. Bye

**Affiliations:** 1 Physiotherapy Department, Royal Prince Alfred Hospital, Sydney, Australia; 2 Sydney Medical School, University of Sydney, Sydney, Australia; 3 Centre for Education & Workforce Development, Sydney Local Health District, Sydney, Australia; 4 Physiotherapy Program, Western Sydney University, Sydney, Australia; 5 Department of Respiratory & Sleep Medicine, Royal Prince Alfred Hospital, Sydney, Australia

**Keywords:** Cystic fibrosis, Body position, Nebuliser delivery rate

## Abstract

**Background:**

Inhalation of nebulised medications is performed in upright sitting to maximise lung volumes. The pattern of deposition is poor for inhaled medications in people with Cystic Fibrosis. The pattern tends to be non-uniform and typically the upper lobes receive a reduced dose compared to the rest of the lung. One strategy that has been proposed as having the potential to improve homogeneity of deposition is to adopt an alternate side lying position for the inhalation procedure. This study sought to determine whether, among adults with Cystic Fibrosis, there is any disadvantage to delivery time of nebulised medications with a strategy of alternate side lying, compared to upright sitting.

**Methods:**

A randomised crossover trial with concealed allocation, intention-to-treat analysis and blinded assessors was undertaken. The participants were 24 adults with stable Cystic Fibrosis. They inhaled 4 mL of normal saline via an LC Star™ nebuliser twice within 24 h. In random order, participants sat upright throughout nebulisation, or alternated between left and right side lying at each minute during the nebulisation period. The nebuliser was stopped and weighed each minute until the residual volume was reached. The primary outcome was the time required for 3.5 mL to be delivered. The secondary outcomes were: respiratory rate; ratio of the volume delivered on right and left sides; and calculation of how long the periods in side lying can be extended without causing greater than 20% discrepancy in dose delivered in the two positions.

**Results:**

The delivery time did not significantly differ between sitting and side lying (mean difference 0.58 min, 95% confidence interval (CI) -1.40 to 0.24). There was no significant correlation between delivery time, lung function or subject height (all R^2^ < 0.4). Increasing side lying duration from 1 to 2 min did not significantly impact the dose delivered on each side. Turning each 3 min however, significantly worsened the disparity (mean ratio 1.32, 95% CI 1.24 to 1.40).

**Conclusion:**

Side lying during inhalation therapy does not prolong nebulisation time. 2-min periods should provide an equal dose in the two side lying positions.

**Trial registration:**

Prospectively registered on 4 July 2011; ACTRN12611000672954.

**Electronic supplementary material:**

The online version of this article (10.1186/s12890-017-0568-2) contains supplementary material, which is available to authorized users.

## Background

Over the past few decades, research has established many nebulised therapies that improve the clinical status and quality of life of people with Cystic Fibrosis, including inhaled tobramycin [1] recombinant human deoxyribonuclease [2] and hypertonic saline [3]. However, these therapies, along with nebulised bronchodilators and other nebulised antibiotics, add considerably to the duration of a patient’s treatment regimen. It is therefore not surprising that prolonged nebulisation times can adversely affect the acceptability of, or full compliance with, treatments for people with Cystic Fibrosis [4].

The distribution of ventilation (V), perfusion (Q), and V/Q matching in the lungs are primarily influenced by gravity [5, 6]. Ventilation is biased to the dependent lung regions regardless of body position. Thus in sitting, tidal ventilation is biased towards basal regions. In healthy subjects and in several patient populations, it has been demonstrated that drug deposition follows the distribution of tidal ventilation within the lungs [7–9]. Nebulised medications are traditionally recommended to be administered in upright sitting to: (a) maximise resting lung expansion in terms of total lung capacity, functional residual capacity, and residual volume [10–12]; (b) maximise ventilation; (c) minimise closing volume of dependent airways; (d) stimulate the sympathetic nervous system to increase alertness and concentration [13] and (e) minimise medication delivery to the oropharynx and gut. However, the disadvantage of the delivery of nebulised therapies in upright sitting is poor deposition of medication in the upper lobes. Poor deposition in the upper lobes has been identified in people with Cystic Fibrosis [7, 14–17], people with HIV [8, 18, 19], and other populations, including healthy people [9, 20, 21].

A strategy of alternate side lying has been proposed as a means to improve upper lobe deposition. In this strategy, patients alternate between right and left side lying at regular intervals during delivery of a single nebulised therapy. The rationale for this approach is that gravity will tend to increase ventilation in the most dependent region of the lungs. Adopting a sidelying position therefore will result in deposition of nebulised medication preferentially throughout the dependent lung, including its upper lobe. Importantly, regular turning is required to dose both sides (including both upper lobes) equally during a single nebulisation period. This is because the dose delivered by an LC Star nebuliser over time is initially rapid and then tapers after 8 to 10 min, rather than constant delivery. This has been demonstrated with its delivery of recombinant human deoxyribonuclease [22] and with its delivery of normal saline in our pilot data (see Additional file 1:. Pilot in-vitro data collected to establish the pattern of decay in the delivery rate of the LC Star nebuliser loaded with 4 mL of normal (0.9%) saline).

It remains unclear, however, whether this alternate sidelying strategy to improve homogeneity of deposition has an impact on nebulisation time. If the nebulisation time was significantly increased then any potential benefits in the pattern of drug deposition may be outweighed by poorer patient compliance in completing the drug administration regimen.

We assert that this is the first study to examine the alternate side lying strategy for nebulised delivery of medication. This assertion is reinforced by a Google Scholar search using the terms *nebuli-* AND *deliver-* AND (*body position* OR *side lying*), which identified no evidence about the side lying strategy.

## Method

### Aims

The aims of the study were to determine:

1. Among adults with Cystic Fibrosis, is there any disadvantage to delivery time of nebulised medications with a strategy of alternate side lying, compared to upright sitting?
2. How long can the periods in side lying be extended without causing greater than 20% discrepancy between the dose delivered in the two positions?

### Design

A randomised, crossover trial with concealed allocation, blinding of assessors and intention-to-treat analysis was undertaken at Royal Prince Alfred Hospital, Sydney. Participants were recruited by personal approach by one of the investigators at the Cystic Fibrosis clinic at the hospital. Once enrolled in the trial, each participant was invited to attend the Department of Respiratory Medicine on two consecutive days. On both days, participants performed spirometry in standing in accordance with the most recent European Respiratory Society criteria [23]. Participants were then randomised, by flipping a coin, to one of two positioning regimens:

1. Upright sitting: maintained throughout the nebulisation period, or.
2. Alternate side lying: alternated between left and right at each minute during the nebulisation period. The starting side was also randomly allocated.

Participants were requested to adopt their allocated position and maintain a slightly deeper than normal tidal breathing pattern during the subsequent standard study inhalation. When participants returned for their second study day, they adopted the other positioning regimen.

All standard morning medications were inhaled on the study days, with the appointment scheduled at least 4 h after these medications, and not within 1 h of a meal. Participants were requested to keep their medication regimen constant during the two study days. After testing on each study day, participants were free to inhale medications and eat as usual.

### Participants

Participants were required to meet the following criteria to be eligible for the study: aged at least 18 years; a diagnosis of Cystic Fibrosis confirmed with sweat testing or genotyping; and clinically stable with a forced expiratory volume in one second (FEV_1_) within 10% of the best recorded value for the past 6 months. Potential participants were excluded if they: had received a lung transplant; were colonised with *Burkholderia cepacia* complex; were not clinically stable; had significant malignant, neurological or musculoskeletal co morbidities; had hepatomegaly, hepatosplenomegaly or current intestinal obstruction; or were pregnant. Research procedures were approved by the Ethics Committees of the Sydney Local Health District (RPAH Zone) (X08-0214, HREC/08/RPAH/358) and the University of Sydney. All participants provided written informed consent prior to participating in this study.

### Intervention

#### Inhalation solution and body position

The standard study inhalation was 4 mL of normal saline (AstraZeneca, North Ryde, NSW), delivered by an LC Star nebuliser (Pari, Germany). The nebuliser and tubing were weighed before and after the 4 mL dose was loaded. The nebuliser was driven with 6 L/min of medical air via the hospital wall supply and a calibrated flow meter. This supply was interrupted after one minute of nebulisation time, as timed with an 870A electronic timer (Diamond Data, China). At this time, the nebuliser and tubing were weighed on a 1206MP Scale (Sartorius, UK). The nebulisation was then recommenced for another timed minute and then weighed again. This continued (with the patient swapping sides during the measurement time if randomised to the alternate side lying condition) until the weight indicated that 3.5 mL of the loaded dose had been delivered from the nebuliser. This approach assumed that the residual volume of 0.5 mL has been reached, in accordance with the manufacturer’s specifications.

#### Blinding

Weighing of the nebuliser was performed by an investigator who was unaware of the position adopted by the participant. This ‘blinding’ was achieved by shielding the investigator from the patient by a curtain and blocking the sound of the participant changing position by padded headphones playing music. The investigator responsible for randomising and positioning the participant (RD) passed the nebuliser and tubing around the curtain to the blinded investigator for weighing each minute. Each weight was recorded until the dead volume has been reached. An Excel file (Microsoft, USA) was used to store the data. The investigator responsible for randomising and positioning the participant recorded the respiratory rate of the participant, counted during the middle 30 s of each minute of nebulisation.

### Outcome measures

The primary outcome was the time required for 3.5 mL of saline to be delivered by the nebuliser as determined by nebuliser weight. The secondary outcomes were respiratory rate, as an explanatory variable; ratio of the volume of saline delivered on the right and left sides; and calculation of how long the periods in side lying can be extended without causing greater than 20% discrepancy between the dose delivered in the two positions, i.e. the ratio exceeding 1:1.2.

### Data analysis

For the primary outcome was nebulised medication delivery time, we were unable to find an estimate of the smallest effect on inhalation time that adults with Cystic Fibrosis would consider using a particular positioning regimen worthwhile. Clinical experience in our centre would indicate that time alone is not the only consideration used by patient for position selection when following a nebuliser regimen. Many patients prefer to nebulise in side lying due to comfort and convenience. Therefore we postulated that the increase in delivery time would need to be substantial to stop the use of this strategy in clinical practice. We considered that a minimum difference of 5 min in delivery time for the 3.5 mL dose would be large enough to contribute to clinical decision-making about which positioning regimen to use. Pilot data provided a standard deviation (SD) of 3.7 min for this change in delivery time among eight adults with Cystic Fibrosis who met the eligibility criteria. Assuming this SD, 20 participants would provide 80% power, at the 2-sided 5% significance level, to detect a 5-min difference in delivery time as statistically significant between two groups in the study. We increased participant recruitment to 24 to allow for possible drop outs.

As an explanatory variable, respiratory rates were compared between sitting and side lying, to see if this variable may have influenced the delivery rate due to breath activation. Respiratory rate in the two delivery positions was compared using a paired t-test. The alternate side lying data were further examined to assess whether the amount of saline delivered while in right side lying differed from that delivered while in left side lying, also using a paired t-test. As noted above, nebuliser delivery rates are not constant, with greater output initially tapering off to low output as the residual volume is approached [22]. To assess whether our strategy of alternating sides during the side lying positioning regimen was successful in balancing the amount of saline delivered in the two side lying positions, the ratio of the amount delivered while on each side was calculated, with a 95% confidence interval (CI). We extrapolated the data to estimate the effect of turning every two minutes, every three minutes, and so on, to identify at what point the amount delivered on each side became unbalanced by greater than 1:1.2.

## Results

### Flow of participants through the study

Twenty-four participants with Cystic Fibrosis were recruited and all completed the study (see Table 1). These participants had characteristics that were representative of the characteristics of patients attending the adult Cystic Fibrosis Clinic at Royal Prince Alfred Hospital in regards to age and lung function (FEV_1_).

**Table 1:** Participant characteristics

	Mean ± SD	Range
Age (yr)	30 ± 9	18–47
Height (m)	1.69 ± 0.08	1.52–1.89
Gender (F: M)	13: 11	
FEV_1_ (predicted %)	55 ± 34	14–108
FVC (predicted %)	76 ± 22	34–122

Mean, standard deviation (SD) and range of characteristics for the 24 participants who completed the study. Forced expiratory volume in 1 s (FEV_1_), Forced vital capacity (FVC)

### Delivery time

**Fig. 1:**
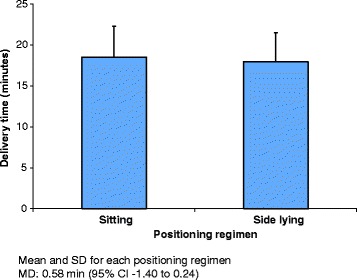
Delivery time in minutes for the two positioning regimens

The delivery time did not significantly differ between the sitting regimen (mean 18.54 ± SD 3.80 min) and the alternate side lying regimen (17.96 ± 3.53 min), a mean difference of 0.58 min (95% CI -1.40 to 0.24) (see Fig. 1).

### Respiratory rate

Respiratory rate did not significantly differ between the sitting regimen (17.9 ± 3.7 breaths / min) and the alternate side lying regimen (17.1 ± 3.8 breaths / min), which was a mean difference of 0.8 breaths / min (95% CI -0.4 to 2.0).

### Volume delivered in right and left side lying

There was no significant difference in the volume delivered in right (1.63 ± 0.14 mL) and left (1.60 ± 0.19 mL) side lying, which was a mean difference of 0.06 mL (95% CI -0.07 to 0.19). Based on extrapolation of the data, increasing side lying duration from 1 to 2 min would not significantly unbalance the dose delivered on each side (see Table 2). To limit the discrepancy between the dose delivered in the two side lying positions to less than 20%, the duration in a position could be extended to 2 min but not to 3 min.

**Table 2:** Ratio of the dose delivered in the two side lying positions

Minute	Ratio	95% CI
1	1.05	0.97 to 1.13
2	1.14	1.09 to 1.19
3	1.32	1.24 to 1.40
4	1.41	1.34 to 1.48
5	1.51	1.40 to 1.61

### Correlations

There was no significant correlation between delivery time and any of the following parameters in either position: FEV_1_, FVC, FEV_1_ % pred, FVC % pred, or participant height (all R^2^ < 0.4).

## Discussion

The delivery times in this study {sitting regimen (mean 18.54 ± SD 3.80 min) and the alternate side lying regimen (17.96 ± 3.53 min)}are consistent with the anticipated delivery rates stated in the product information for an LC Star and observed in previous work in our centre. Clinicians may note that this is a longer duration than is often observed for the delivery of 4 mL of normal saline in clinical practice. We believe that this is because some patients only nebulise until the first sign of intermittent delivery, rather than continuing until the dead volume has been reached. The time burden of inhalation therapy supports the need to find strategies to improve convenience for individuals with cystic fibrosis.

Alternate side lying does not slow the delivery of nebulised medications substantially. The best estimate is that delivery of 3.5 mL of medication is just over half a minute faster, which would make up for the time spent turning, making it time neutral on average. This finding generates implications for further research. The time-neutral nature of the alternative side lying regimen indicates that it is suitable for further investigation of its effect on medication deposition pattern. This research could be pursued not just in people with Cystic Fibrosis but also in the other diseases where poor upper lobe deposition has been identified during delivery of nebulised therapies in upright sitting [8, 9, 18–21].

Turning between right and left side lying each minute delivered an equivalent dose by weight while in each side lying position. However, turning this frequently is likely to be impractical in clinical practice. Based on extrapolated data, increasing side lying duration from 1 to 2 min would not significantly unbalance the dose delivered on each side, but any further extension of time (≥ 3 min) would cause medication delivery imbalance. It is possible that patients with Cystic Fibrosis would choose to adopt a single side to nebulise for a therapy session and then lie on the other side for the next session. Alternatively, they may spend half the delivery side on one side and turn to the other side for the remainder, but alternate the starting side for subsequent doses. However, investigation of the side lying regimen’s effect on the pattern of drug deposition is needed before it can be recommended in clinical practice. Therefore we do not recommend that the results of this study be used to generate any immediate clinical implications about positioning regimens during nebulised delivery of medications for people with Cystic Fibrosis. However, this study will be useful to inform the design of further studies about the effects of body position on nebulised drug delivery. Subsequent future work could then consider the impact of body position in relation to faster delivery systems and other inhaled therapies (dornase alfa, antibiotics) with differing viscosities.

## Conclusions

The use of an alternate side lying positioning strategy during nebulised therapies does appear to be suitable, from the perspective of nebuliser delivery time, as an alternative to upright sitting. However while total nebuliser dosage delivered while in each side lying position does appear to be equitable if the side is changed at least every 2 min, further investigation of the side lying regimen’s specific effect on the pattern of drug deposition is needed before this position can be recommended.

## Additional file

Additional file 1: Pilot in-vitro data collected to establish the pattern of decay in the delivery rate of the LC Star nebuliser loaded with 4 mL of normal (0.9%) saline. Datasets are available from the first named author (R Dentice) on reasonable request. (DOCX 16 kb)

## Abbreviations

CI: Confidence interval
FEV_1_: Forced expiratory volume in one second
FVC: Forced vital capacity
Q: Perfusion
SD: Standard deviation
V: Ventilation
