# Supplementary material for: The use of an alternate side lying positioning strategy during inhalation therapy does not prolong nebulisation time in adults with Cystic Fibrosis: a randomised crossover trial
Source: BMC Pulm Med. 2018 Jan 8;18:3. doi: 10.1186/s12890-017-0568-2 (PMC5759805; doi:10.1186/s12890-017-0568-2)
Supplement: Additional file 1: — Pilot in-vitro data collected to establish the pattern of decay in the delivery rate of the LC Star nebuliser loaded with 4 mL of normal (0.9%) saline. Datasets are available from the first named author (R Dentice) on reasonable request. (DOCX 16 kb) [file 12890_2017_568_MOESM1_ESM.docx]

Additional file **1**

**Pilot in-vitro data collected to establish the pattern of decay in the delivery rate of the LC-Star nebuliser loaded with 4mL of normal (0.9%) saline.**

Aim: To generate pilot data about the pattern of change in the delivery rate of the LC-Star nebuliser during the delivery of 4mL of normal saline (0.9%), in order to inform the design of the submitted study.

Methods: One LC-Star was clamped upright on a benchtop and was used to nebulise 4mL of normal saline (0.9%), running on medical air at 6 L/rain. The nebuliser was weighed before and after the saline was loaded, and after each minute of the nebulisation period. The nebuliser has a "dead volume" – that is, a residual volume that does not reliably nebulise – of 0.5mL. Therefore, nebulisation continued until the weight of the nebuliser indicated that the dead volume had been reached. Calculation from weight to volume was based on the conversion 1 g = 1 mL.

Results: Visual inspection of the figure indicates that the delivery rate is not constant. The delivery rate was initially 0.34 mL/min from 0 to 8 minutes. The delivery rate then slowed to 0.18 mL/min between 8 and 12 minutes. The delivery rate then slowed further to 0.05 mL/min between 12 and 14 minutes. The dead volume was reached between 14 and 15 minutes.

Conclusions: The LC-Star nebuliser does not have a stable delivery rate throughout the delivery. The delivery rate slows as the remaining volume to be nebulised approaches the dead volume of the nebuliser.
